# Insect Brains Use Image Interpolation Mechanisms to Recognise Rotated Objects

**DOI:** 10.1371/journal.pone.0004086

**Published:** 2008-12-31

**Authors:** Adrian G. Dyer, Quoc C. Vuong

**Affiliations:** 1 Department of Physiology, Monash University, Clayton, Victoria, Australia; 2 Institut fur Zoologie III (Neurobologie) Johannes Gutenburg Universität, Mainz, Germany; 3 Institute of Neuroscience, Newcastle University, Newcastle upon Tyne, United Kingdom; University of Southern California, United States of America

## Abstract

Recognising complex three-dimensional objects presents significant challenges to visual systems when these objects are rotated in depth. The image processing requirements for reliable individual recognition under these circumstances are computationally intensive since local features and their spatial relationships may significantly change as an object is rotated in the horizontal plane. Visual experience is known to be important in primate brains learning to recognise rotated objects, but currently it is unknown how animals with comparatively simple brains deal with the problem of reliably recognising objects when seen from different viewpoints. We show that the miniature brain of honeybees initially demonstrate a low tolerance for novel views of complex shapes (e.g. human faces), but can learn to recognise novel views of stimuli by interpolating between or ‘averaging’ views they have experienced. The finding that visual experience is also important for bees has important implications for understanding how three dimensional biologically relevant objects like flowers are recognised in complex environments, and for how machine vision might be taught to solve related visual problems.

## Introduction

The ability to reliably recognise three dimensional objects is a complex problem for both biological and artificial vision systems since the viewpoint from which the object is seen may dramatically affect the spatial relationships between visible local features of the object [1]–[4]. Many biologically important objects like flowers for bees [5], or faces for primates [6], sheep [7] and even wasps [8], [9], have to be viewed in complex natural environments from different viewpoints. This can be a particularly difficult problem for visual systems to solve as an image of a rotated target stimulus, like a face, will often appear more dissimilar to its non-rotated appearance than to other non-rotated distractor stimuli [3].

Adult humans[10], [11] and other primates[6], [12], [13] recognise novel presentations of rotated objects through mechanisms that predominantly rely on image interpolation of a limited number of stored views. In the primate brain, for instance, neurons in inferior-temporal cortex can become tuned to trained views of objects [6], [13], [14]. The response of these neurons to stimuli gradually decreases depending on how similar a novel view is to the neurons' preferred view [14]. There is some evidence that neurons further upstream in inferior-temporal cortex accumulate responses from the population of view-tuned neurons, and thus by summing the responses across different view-tuned neurons the visual system may average across stored views to recognize novel views [13]. This kind of averaging can be implemented by a biologically plausible radial basis function network comprised of an input, an output and a hidden layer which learns a smooth function to interpolate novel views between stored views [15]. Interestingly, primate brains perform better with novel views that fall within stored views (interpolations) than with those that fall outside stored views (extrapolations) [10], but some other animal models, such as pigeons, respond equally well to both interpolated and extrapolated views [16].

Recent studies on the processing of visual stimuli by honeybees suggest that their miniature brains can accomplish relatively sophisticated visual tasks [17]–[20], in a manner that may point to efficient processing algorithms [20], [21]. Furthermore, when provided with differential conditioning, bees show a remarkable ability to learn complex stimuli utilizing global cues [17], [19], [22], [23]. To understand how miniaturized brains might deal with the problems posed by rotations in depth, we presented bees with a face recognition task that has recently been useful for evaluating face processing in infant humans [3]. This procedure allows for testing with complex but reasonably homogeneous stimuli set for which individual bees can have no specific ontogenetic experience [24]. A key question in understanding how brains recognise stimuli when viewed from different viewpoints is whether a brain transforms a stored representation, or does a brain enable recognition to occur as a consequence of interpolation between learned views [14]. Moreover, by using a stimulus set that has been useful for understanding infant vision; some inferences might be possible about how brains from remarkably different phylogenetic backgrounds solve the task of recognising novel rotated views of previously learnt stimuli. We chose to use faces as stimuli in the current study, rather than more biologically-relevant stimuli such as flowers, to be certain that the bees had no prior experience with the stimuli. Furthermore, the human studies suggest that image interpolation mechanisms are generic in the sense that they operate over both biologically relevant and irrelevant stimuli.

## Results and Discussion

**Figure 1:**
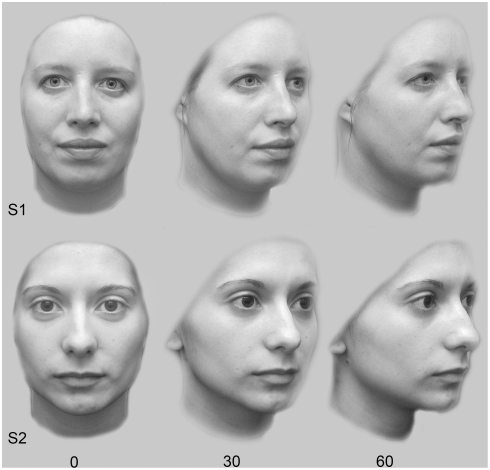
Rotated face stimuli used to train bees with differential conditioning.

Individual honeybees (*Apis mellifera*) were trained with differential conditioning to stimuli [17], [24] representing different views (0°, 30° or 60°) of two similar faces (S1, S2; Fig. 1). These same stimuli have been used for understanding face processing in newborn humans [3], and as face stimuli are novel for bees it is possible to collect data on how a brain with no previous experience with the stimulus set solves the task. Group 1 was trained with face images at a 0° view, and then given non-rewarded tests with these stimuli and novel 30° stimuli. Group 2 was trained with 60° stimuli and tested with these stimuli and with novel 30° stimuli. Group 3, our critical group, was provided training with both the 0° and the 60° view before being tested with these stimuli and novel 30° interpolation stimuli. Finally, Group 4 was trained with both the 0° and the 30° stimuli and tested with novel 60° extrapolation stimuli. In each group, the target-distractors (S1/S2) were reversed for half the bees to control for potential preference effects [19].

**Figure 2:**
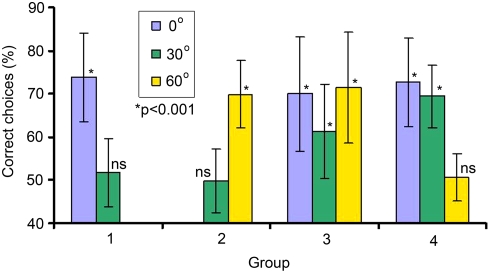
Mean frequency of correct choices (±s.d.) for honeybees recognising images of rotated face stimuli. Bees in Groups 1 and 2 could not recognise a novel view of the target different from chance performance (50%), but bees in Group 3 could recognise a novel 30° view (by interpolating 0° and 60° images). Bees in Group 4 could not recognise a novel presentation of 60° by extrapolating from learnt 0° and 30° views. For non-significant results (ns) p>0.35.

Following differential conditioning, bees in all four groups were able recognise the trained target stimuli significantly above chance performance (Fig. 2). This new finding shows that the bees in Groups 3 and 4 can, in addition to storing one complex spatial pattern like a face [24], store and successively retrieve multiple views of a complex shape. When these highly trained groups of bees were presented with a novel view of the target and distractor face stimuli, only bees in Group 3 were able to recognise the correct face significantly above chance (Fig. 2). However, even for group 3, there was some level of disruption to the visual processing as the performance was poorer than for the recognition of the original training stimuli (paired sample t-test, t = 3.419, df 29, p = 0.002). The target recognition by bees in Group 3 cannot be explained by generalization principles [25], as bees in neither Group 1 nor Group 2 were able to recognise a novel view of the target face (Fig. 2). The data thus imply that bees in Group 3 were able to extract some relevant information from conditioning with both 0° and 60° views, consistent with the idea of image interpolation [10], [15].

Another possibility that could explain how bees in Group 3 were able to recognise a novel presentation of the target stimulus is that learning multiple representations of the stimuli might promote greater flexibility to solve a novel task [20], [26]. However, this explanation can be excluded as bees in Group 4 were not able to recognise a novel view at 60° (Fig. 2). Thus, neither a form of image extrapolation nor increased neural flexibility for problem solving can explain how bees in Group 3 were able to recognise a novel view of the target stimulus, strongly suggesting that the recognition must solely be due to a mechanism of image interpolation. Future experiments may consider the role of experience with a variety of different viewpoints on the ability of insect brains to solve complex spatial tasks like image rotation of either face or biologically relevant stimuli.

Like many animals that operate in complex visual environments, bees have to reliably find three dimensional objects like flowers when these objects might be seen from a number of different views [5]. In primates there is evidence that recognising objects independent of viewpoint is solved both through innate mechanisms present from birth [3], [27], and also experience at viewing stimuli from a variety of different viewpoints [10], [12], [15]. In this study we have been able to show that an invertebrate brain can learn to reliably recognise the stimuli with experience by using a mechanism of image interpolation. However, unlike primates or pigeons, bees rely more strongly on image interpolation mechanisms than other species, in that they are unable to recognize extrapolated views whereas primates and pigeons can. These species differences may point to different implementations of image interpolation mechanisms, given the large anatomical differences among species. For example, bees may have a much narrower view tuning relative to the higher vertebrates, which may limit their capacity to generalize from a single view (Groups 1 and 2) or to extrapolated views (Group 4). Despite these species differences, the overall findings are consistent with view-based models of object recognition [10], [15]. A central idea of this class of models is that specific views of objects are represented, which encode features under specific viewing conditions such as viewpoint, rather than view-invariant features such as three-dimensional structure [28]. This finding is consistent with data for how animals with much larger brains learn to reliably recognise novel rotated objects by interpolating previously learnt views, and thus supports the ideas that simple networks could recognise three dimensional objects by interpolating between relatively small numbers of previously learnt views [6], [14], [15].

## Materials and Methods

### Behavioural testing

**Figure 3:**
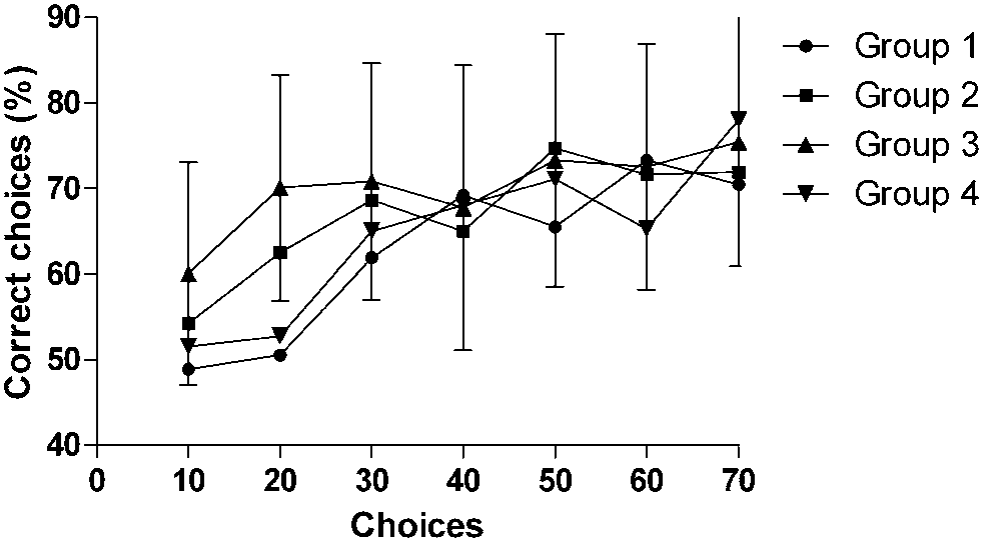
Acquisition (N = 18 bees for Groups 1, 2 and 4; N = 30 bees for Group 3 showing mean±s.d.) for bees learning with differential conditioning to recognise target from distractor stimuli (images of similar human faces). Group 1 learnt only stimuli at 0° angle of view, Group 2 only at 60° angle of view, Group 3 learnt both 0° and 60° angles of view, and Group 4 learnt both 0° and 30° angles of view.

Experiments were conducted outdoors in fine weather conditions. Honeybees were recruited from a gravity feeder [29] providing 10% sucrose, and rewarded with 25% sucrose for making correct choices on designated target stimuli presented vertically using a rotating screen of 50 cm diameter [17], [24]. This screen presents the face stimuli on hangers so that the spatial position of stimuli can be continuously changed during training (to exclude bees using stimulus position as a cue). The screen also enables collection of data by counting choices (touches to the landing stage of stimuli) which are not dependent on the actual visual angle at which a bee chooses to view the stimuli prior to making a decision [24]. A photograph of the rotating screen is presented in a previous study [24]. Each bee was tested individually, typically taking 6–7 hours. Incorrect choices (landings on distractor stimuli) were punished with 0.012% quinine hemisulphate which leads to a very high level of motivation in bees to perform tasks well [30]. Stimuli were 6×8 cm achromatic photographs made from image files supplied by Dr Turati from a study that used these stimuli for investigating face rotation processing in newborn humans [3]. In the current study the face images were presented on a light grey background (Fig. 1) as pilot tests indicated that bees were distracted by a high contrast black background. Two identical target and two identical distractor stimuli were presented on the screen at one time, promoting differential conditioning which forms a long term memory that will persist for at least two days [23], [24] and promotes global learning of local features [17], [19], [22], [23]. Bees were provided with a 10 µL drop of sucrose for a correct choice, and a second drop was presented on a plexiglas spoon [24] to move the bee 1 m away so that fresh stimuli could be exchanged during training. When the bee became satiated, it returned to the colony and all equipment was cleaned with 30% ethanol. For Groups 3 and 4, training was with different views of the target and distractor stimuli in alternative bouts. Each bee was trained until it met the precondition of correctly choosing the target stimulus with >50% accuracy in six consecutive bouts, and Figure 3 shows the mean acquisition for the bees in the different groups during learning. Because of the precondition some bees received training for longer than 70 landings prior to the non rewarded testing, but a similar level of target stimulus recognition was achieved by the 4 different groups after 70 stimulus visits (Fig. 3) suggesting that learning these different but difficult visual tasks places somewhat similar levels of demand on the visual system of the bee. Interestingly, a similar level of slow acquisition for difficult tasks has been previously reported for bees learning both natural scenes [17], and complex artificial stimuli [19], [31]. Once the precondition was met, a bee was provided with a non-rewarded test with fresh versions of the training stimuli (to totally exclude olfaction), followed by refresher bouts with training stimuli (for motivation), and a second non-rewarded transfer test with novel stimuli. Finally, a bee was retested with the initial stimuli to confirm possible performance drops were not due to temporal factors (statistical tests for this possibility revealed no temporal factors during testing affected bee performance). All testing with each individual bee was completed within one day.

### Statistical analysis

Bees learnt the visual task slowly (Fig. 3), consistent with previous reports on how bees learn difficult visual problems [17]–[19], [24]. All data used for statistical analysis is from non-rewarded bouts following training. Discrimination of learnt targets following differential conditioning were statistically tested using a one-sample t-test on arcsine square-root transformed proportions with sequential Bonferroni correction (p-value set to 0.0045, two tailed tests) for multiple comparisons [Group 1, (0°) = 73.8% (10.2 s.d.), t = 7.169, N = 18, df = 17, p<0.001; Group 2, (60°) = 69.9% (7.9 s.d.), t = 9.119, N = 18, df = 17, p<0.001; Group 3, (0°) = 70.0% (13.3 s.d.) t = 7.795, N = 30, df = 29, p<0.001; Group 3(60°) = 71.5% (12.8 s.d.) t = 8.647, N = 30, df = 29, p<0.001; Group 4, (0°) = 72.7% (10.3 s.d.), t = 8.667, N = 18, df = 17, p<0.001; Group 4, (30°) = 69.4% (7.3 s.d.), t = 10.77, N = 18, df = 17, p<0.001].

Following the refresher training, discrimination of learnt faces from novel views was evaluated in non-rewarded tests in a similar manner. Group 1 (30°) [51.7% (7.9 s.d.), t = 0.947, df 17, p = 0.357], Group 2 (30°) [49.8% (7.4 s.d.), t = 0.920, df 17, p = 0.928], Group 3 (30°) (61.4% (10.9 s.d.), t = 5.472, df 29, p<0.001; performance for a subset of first 18 bees tested was 59.6% (8.8 s.d.), t = 4.546, df 17, p<0.001), Group 4 (60°) [50.6% (5.4 s.d.), t = 0.438, df 17, p = 0.667] (Fig. 2). Comparing the subset of data for groups 3 and 4 were also significantly different (independent samples t-test on arcsine square-root transformed proportions, t = 4.684, df 34, p<0.001).
